# Cutting Forces Measurement for Milling Process by Using Working Tables with Integrated PVDF Thin-Film Sensors

**DOI:** 10.3390/s18114031

**Published:** 2018-11-19

**Authors:** Ming Luo, Zenghui Chong, Dongsheng Liu

**Affiliations:** Key Laboratory of Contemporary Design and Integrated Manufacturing Technology (Northwestern Polytechnical University), Ministry of Education, Xi’an 710072, China; chongzenghui@163.com (Z.C.); 2014200870@mail.nwpu.edu.cn (D.L.)

**Keywords:** cutting force, PVDF sensor, milling

## Abstract

In the milling process, cutting forces contain key information about the machining process status in terms of workpiece quality and tool condition. On-line cutting force measurement is key for machining condition monitoring and machined surface quality assurance. This paper presents a novel instrumented working table with integrated polyvinylidene fluoride (PVDF) thin-film sensors, thus enabling the dynamic milling force measurement with compact structures. To achieve this, PVDF thin-film sensors are integrated into the working table to sense forces in different directions and the dedicated cutting force decoupling model is derived. A prototype instrumented working table is developed and validated. The validation demonstrates that profiles of the forces measured from the developed instrumented working table prototype and the dynamometer match well. Furthermore, the milling experiment results convey that the instrumented working table prototype could also identify the tool runout due to tool manufacturing or assembly errors, and the force signal spectrum analysis indicates that the developed working table can capture the tool passing frequency correctly, therefore, is suitable for the milling force measurement.

## 1. Introduction

Cutting force, which is originated by the shearing of the material, friction between the chip and the cutter and so on, can convey key information on the conditions of machining processes. The cutting forces can reflect the machinability of the material [1,2] or used to identify machining malfunctions, such as machining vibrations [3,4] or tool wear [5]. Therefore, measuring cutting forces in the machining process is fundamental for condition monitoring and process optimization [6,7]. The commonly used method for cutting force monitoring is the application of a dynamometer, which is stable, accurate and has good repeatability [8]. However, since the dynamometer is usually large and heavy, it cannot be directly assembled in a simple way for continuously measuring forces in a workshop. Therefore, the measuring of cutting forces in the machining process with embedded sensors or integrated into the instrumented fixtures and tools is a promising way which has little effect on the existing machining environment. For this to happen, some instrumented table/structure or instrumented tools with embedded sensors have been developed in recent years [9].

Instrumented tables or components are designed to integrate sensors or to be used as sensors to measure forces and vibrations. Zhao et al. [10] developed a structure with two mutual-perpendicular octagonal rings and three Wheatstone full bridge circuits in order to obtain the tri-axial cutting force components and restrain cross-interference in turning process. Yu et al. [11] proposed a flexible piezoelectric tactile sensor array based on a polyvinylidene fluoride (PVDF) film for measuring three-axis dynamic contact force distribution. Li et al. [12] presented a strain-type three-component table dynamometer with has a sensor structure with eight parallel elastic beams and sensitive regions and Wheastone measuring circuits are designed to eliminate the influence of eccentric forces. To monitor the machining process, Shi and Gindy [13] integrated the strain sensor, accelerometer and power sensor into the machine tool to detect tool malfunctions for machining processes. Luo et al. [14] embedded PVDF thin-film sensors into the fixtures to monitor the vibration of thin-walled components during the flank milling process. Li et al. [15] developed a responsive fixture by integrating the piezoelectric pressure sensor and eddy current displacement sensor into the fixture to ensure the precision machining of large-scale aerospace parts. Wan et al. [16] integrated a strain gauge into the workpiece to measure the magnitude of force transformed by the fixture, which is used to improve the machining stability.

To further measure cutting forces acting on the cutting tool in the machining processes, developing instrumented cutting tools is an alternative method for understanding the cutting process. Wang et al. [17] presented a lathe tool with two surface acoustic wave strain sensors mounted onto the top and side surface of the tool shank to measure cutting force and feed force. Nguyen et al. [18] used the PVDF strain rosette to monitor dynamic cutting forces and torque in single-point cutting processes, specifically turning and boring. The above studies are wired method for cutting forces measurement, which cannot be used in rotation cases. To measure cutting forces in rotating cases, wireless instrumentation methods are also developed. Totis et al. [19] developed a rotating instrumented cutter by integrating a tri-axial force sensor into the cutter for cutting force measurement in face milling. Liang et al. [20] developed a six-component sensor system with a compact monolithic elastic element to detect the tangential cutting forces as well as the cutting moments simultaneously in the machining processes. Ma et al. [21] used PVDF sensors to pick up the dynamic shear strain produced in the rotating tool during the cutting process. Luo et al. [22] integrated the PVDF thin-film sensors into the index-able tools to measure cutting forces acting on separate insert, the signals are transmitted by a wireless transmitter, and the signals are then used to identify the insert working condition in the milling process. Liu et al. [23] designed an integrated rotating dynamometer based on fiber Bragg grating (FBG) to measure four-component cutting force. These methods mainly integrate sensing components into the cutting tool, however, this is easy to be disturbed by the machining process since rigidity of the spindle is relatively low.

Machining process monitoring is an emerging requirement in the Industry 4.0 era, where the concept of integrating sensors into machine tools is a promising way that will not have a large influence on existing production lines. To this end, an instrumented working table with embedded PVDF thin-film sensors was developed and verified in this study. The main contribution of this paper is the usage of PVDF sensor to pick up the dynamic normal strain from an instrumented table for milling force measurement. The design conception of the instrumented working table and the PVDF based cutting force sensing method are presented in Section 2, the cutting force decoupling model is developed in Section 3. The experimental validation results and discussion are given in Section 4, followed by conclusions in Section 5.

## 2. Conceptual Designs for Instrumented Working Table

### 2.1. The Design Conception

**Figure 1:**
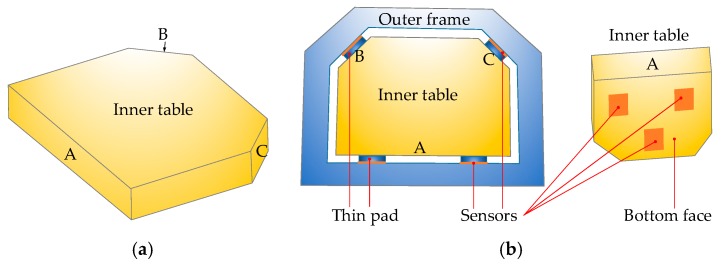
The design conception for the instrumented working table with integrated sensors. (**a**) Inner table, (**b**) assembled table.

In the milling process, cutting forces are applied directly on the workpiece as well as the connecting holding table. To measure these cutting forces, the basic idea is to put sensors around the table to sense forces in all three directions: *X*, *Y* and *Z*. However, to fix the working table in terms of translation and rotation, in total there should be six locators with sensors. Besides, since the bottom face is relatively large, three sensing locations are needed. To sense the cutting forces in the milling process, within the *X*-*Y* plane, sensors are placed on face A, B and C, which are also faces contacting with the outer frame. Based on the above analysis, the conceptual design of the instrumented working table is shown in Figure 1. On face A, two sensors are used since it is relatively large and rotation may occur if only one locator is used. With this design, forces within the *X*-*Y* plane can be sensed by the sensor. In the *Z* direction, there are three sensors on the bottom face and they will sense force component in the *Z* direction.

### 2.2. PVDF Based Cutting Force Sensing

#### 2.2.1. Cutting Force Sensing System with PVDF Sensors

**Figure 2:**
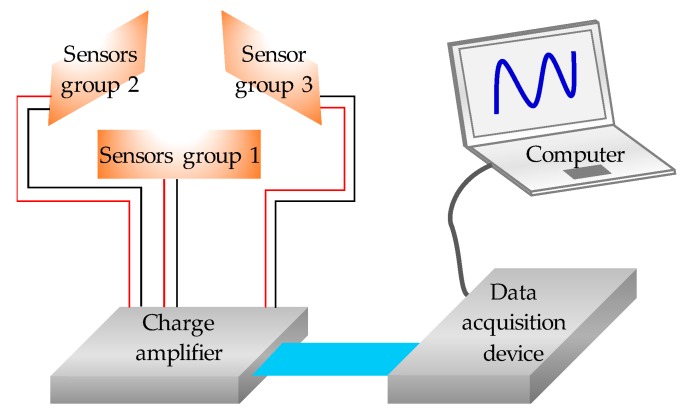
The schematic diagram for the cutting forces sensing and recording.

Since the milling process is a highly interrupted process and forces change with the instantaneous chip thickness, cutting forces in all tangential, radial and axial directions vary fast with the progress of cutting. Therefore, sensors with high working frequency range should be employed. To this end, the commercially available PVDF thin-film sensors with connecting wires are used in this study since it is highly flexible and has a high working frequency up to 10 MHz. Each PVDF sensor has a size of 10 × 10 mm and a thickness of 50 μm. Once the PVDF sensor deforms under the external force, an equal quantity of charges on the surfaces of electrodes will be induced by polarized charges insides of the thin film. These charges are then measured by a charge amplifier and output as voltage, which is then collected by the data acquisition system for further analysis. Three groups of PVDF sensors are used in this study and the basic concept for the cutting forces sensing is shown in Figure 2.

#### 2.2.2. Charge Calculation for PVDF Sensors

**Figure 3:**
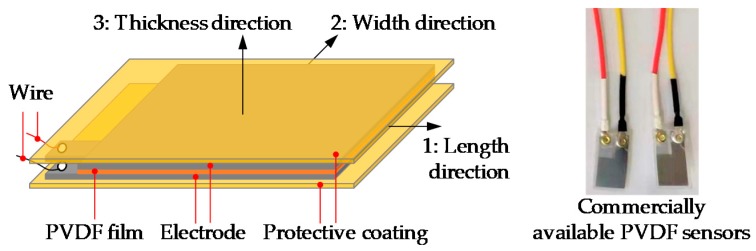
Schematic configuration of the PVDF thin-film sensor.

As shown in Figure 3, a PVDF sensor has a multilayer sandwich structure consisting of a PVDF thin-film, two electrodes, the whole structure is wrapped by coatings for protection and insulation. The total thickness of the sensor can vary from 10 μm to hundreds of micrometers according to its application. When an external force component is applied on the PVDF sensor, the electric charges generated by the mechanical strains of the sensor can be computed by integrating the electric displacements D over the electrode area as follows [24]:

(1) $$\;q=\iint \left[\begin{array}{ccc} D_{1} & D_{2} & D_{3} \end{array}\right]\left[\begin{array}{c} dA_{1}\\ dA_{2}\\ dA_{3} \end{array}\right]\;$$

where $dA_{1}$, $dA_{2}$ and $dA_{3}$ are the components of the electrode area in the 2-3, 1-3 and 1-2 planes respectively, as shown in Figure 3.

Since the thickness of the PVDF sensor is very small and the PVDF element of the sensor can be treated as orthotropic material after being poled, the sensor can be assumed to be in a state of plane stress. Therefore, if the force component is applied only on the normal face of the sensor, the strain mainly exists in the normal direction, the shear strain caused by torsion or slide is neglected here since the PVDF sensor is insensitive to the in-plane shear strain. Hereby, the charged induced in element area $dA_{i}$ can be derived as:

(2) $$\;q_{i}=d_{33}\sigma_{i}dA_{i}=d_{33}dF_{i}\;$$

where $d_{33}$ is the piezoelectric constant in the 3 direction, and the stress $\sigma_{i}$ can be written as [25]:

(3) $$\;\sigma_{i}=dF_{i}/dA_{i}\;$$

Since the working table is usually large, more than one PVDF sensor should be used for force sensing in one direction, which requires a parallel array of thin film sensors. In this case, the output charge will be the sum of all sensors.

#### 2.2.3. Forces Calculation Based on Generated Charges

To record signals induced by the cutting forces, the output charge is converted to voltage by a charge amplifier and then filtered by a filter circuit. Once connected to the circuit of charger amplifier, the output voltage can be expressed as [26]:

(4) $$\;V=-q/C_{f}\;$$

where $C_{f}$ is the electric capacitance of the circuit feedback loop, it can be calibrated by experiments. If the pressure over the sensor is evenly distributed, the output voltage is related to the applied force F by the relation:

(5) $$\;V=\frac{d_{33}}{-C_{f}}F\;$$

In this paper, the applied force on PVDF sensor is the dynamic cutting force which has an higher frequency than the lower cut-off frequency of the charge amplifier circuit. Thereby, the applied force can be calculated by the above equation.

## 3. Cutting Force Decoupling Model

In order to utilize the proposed concept, the decoupling of forces based on the monitored signals with PVDF sensors is required. Since the table is used for fixing the workpiece and will not move during the machining process, the forces acting on the table are balanced.

### 3.1. Synthesis and Decomposition of Forces

Consider a solid body with n forces $F_{1},\;F_{2},\cdots ,F_{n}$ applying on it within a plane, while applying the translation theorem of force, moving these forces to a center O, then forces $F_{1}^{\text{’}},F_{2}^{\text{’}},\cdots ,F_{2}^{\text{’}}$ with corresponding force couples can be obtained. In this way, any force on the plane is equivalent to two simple force systems: the planar force and the planar force couple. Since $F_{i}^{\text{’}}=F_{i}$, and the planar force system can be synthetized into a force $F_{R}^{\text{’}}$:

(6) $$F_{R}^{\text{’}}=F_{1}^{\text{’}}+F_{2}^{\text{’}}+\cdots +F_{n}^{\text{’}}.\;$$

That is, $F_{R}^{\text{’}}$ is the vector sum of the original applied forces.

The planar force couple system can be synthesized as a couple, and the moment $M_{O}$ of the couple is equal to the algebraic sum of the additional moments and the algebraic sum of the moments of the original forces to the point O, which is:

(7) $$\;M_{O}=M_{1}+M_{2}+\cdots +M_{n}\;$$

The vector sum $F_{R}^{\text{’}}$ of all forces in a coplanar arbitrary force system is called the principal vector of the force system; and the algebraic sum of moments for these forces referring to an optionally reduction center O is $M_{O}$, and it is the principal moment of the force system for the reduction center. Obviously, the principal force vector is independent of the reduction center, and the principal moment is generally related to the reduction center, so it is necessary to specify the principal moment for which point the force system is.

When a force system satisfies its principal vector and the principal moment of any point is zero, it can be obtained that the coplanar arbitrary force system is balanced. That is, when performing the cutting force measurement, the combined forces are respectively obtained for the three directions of X, Y and Z. If it can be ensured that the combined force of the main body of the table in each of the three directions is zero, the working table is in an equilibrium state. Since the table is restricted and does not move during the machining process, it is in equilibrium state. Therefore, the measurement of the cutting force is converted into the pressure change of the piezoelectric sensors.

### 3.2. Calculation of Forces in X and Y Directions

**Figure 4:**
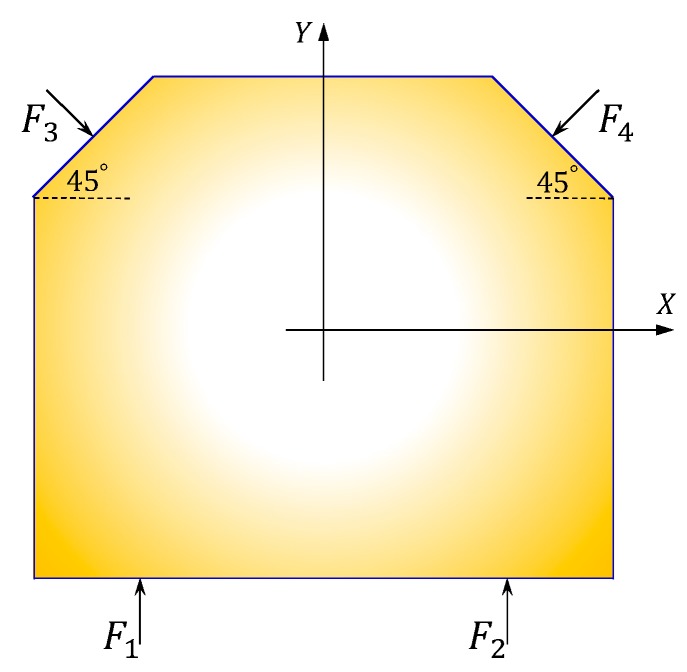
Forces in the X and Y directions.

In the milling process, four sensors placed on the faces A, B and C to sense the cutting forces in the X-Y plane, as shown in Figure 4. Since the table cannot move, all forces applied on the table should be balanced. Then the following Equations hold

(8) $$\;\{\begin{array}{c} \overset{n}{\underset{i=1}{\sum }}F_{xi}=0\\ \overset{n}{\underset{i=1}{\sum }}F_{yi}=0 \end{array},\;i=1,2,3,4\;$$

Forces in the X and Y directions can be derived as

(9) $$\;\{\begin{array}{l} F_{x}=\frac{\sqrt{2}}{2}F_{3}-\frac{\sqrt{2}}{2}F_{4}\\ F_{y}=F_{1}+F_{2}-\frac{\sqrt{2}}{2}F_{3}-\frac{\sqrt{2}}{2}F_{4} \end{array}\;$$

where $F_{i}\;\left(i=1,2,3,4\right)$ is the force component sensed by the corresponding sensor.

### 3.3. Calculation of Force in Z Direction

**Figure 5:**
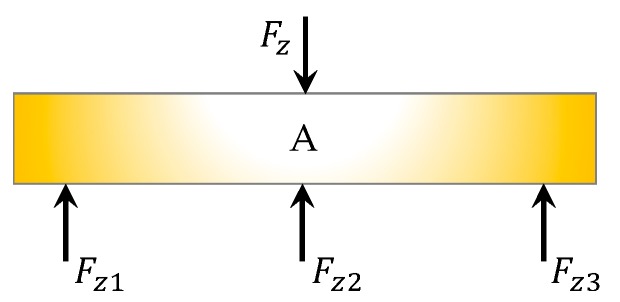
Forces in the Z direction.

As shown in Figure 5, on the bottom face of the table, there are three sensors used for forces sensing in the Z direction. Since sensors on other planes do not have components in the Z direction, there is no coupling in the Z direction, and the force can be expressed as

(10) $$\;F_{z}=F_{z1}+F_{z2}+F_{z3}\;$$

## 4. Experimental Validation and Discussion

To verify the accuracy of the developed monitoring platform, both the cutting force signals monitored by the instrumented working table and the dynamometer will be recorded and compared in this section.

### 4.1. Realization of Instrumented Working Table

**Figure 6:**
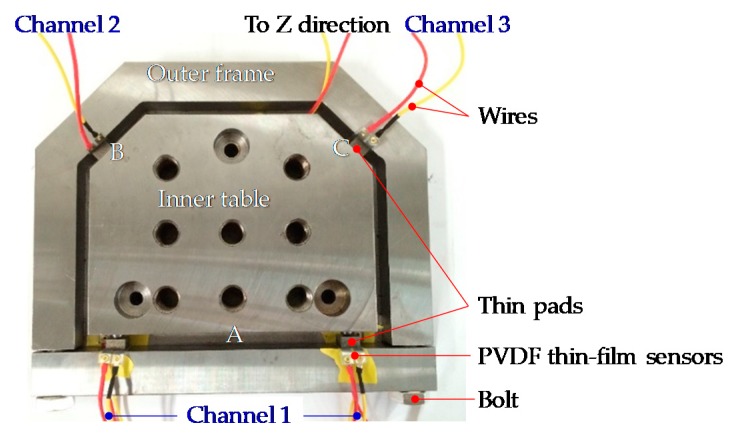
The assembled instrumented working table prototype.

To validate the proposed concept, a working table with embedded PVDF sensors was manufactured and assembled, as shown in Figure 6. The prototype table is made of 45 steel, and preloading is applied on the sensors in the assembly process. A multi-channel charge amplifier is connected to the PVDF sensors, it is then connected to a DEWEsoft data acquisition system for signal recording. The piezoelectric constant *d*_33_ of the PVDF sensor is 43.94 pC/N, the sensitivity of the charge amplifier is 0.1 mV/pC, its working frequency range is from 0.8 Hz to 10 kHz. The collected charge signal can be directly converted into the cutting force output through the setting in the data acquisition system.

To validate the proposed prototype, a 9255B table dynamometer (Kistler, Winterthur, Switzerland) is used to the record forces for comparison. The workpiece is fixed on the instrumented table, the table is then fixed on the dynamometer.

### 4.2. Experimental Set-Up

**Figure 7:**
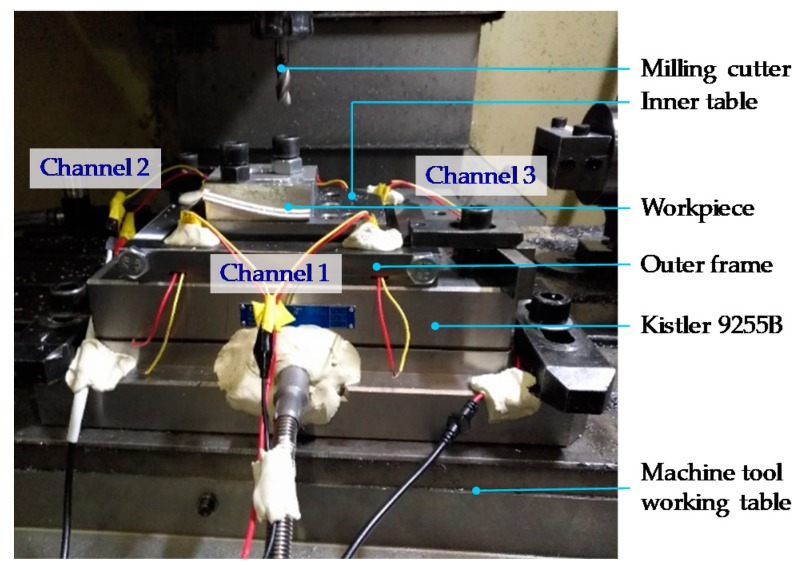
The experimental setup.

The material used for validation is aluminum alloy, the cutter is a 10.0 mm diameter HSS flat-end milling cutter with three flutes. The developed instrumented table is fixed on the top of a Kistler 9255B dynamometer, a SA-PE03 charge amplifier (Shiao, Wuxi, China) and a STG data acquisition device (DEWESoft SIRIUSi-SYSTEM, Trbovlje, Slovenia) are connected to the table. Dry cutting condition is applied in the milling test to avoid the influence of cutting fluid on cutting forces and to avoid the damage to the developed monitoring system. To validate the instrumented working table, a certain cutting parameters range with axial and radial cutting depth varies from 1 to 3 mm are applied, and the spindle speed varies from 2000 rpm to 4000 rpm. The experimental setup is shown in Figure 7, and the cutting parameters are shown in Table 1.

### 4.3. Results and Discussion

#### 4.3.1. Cutting Forces

**Figure 8:**
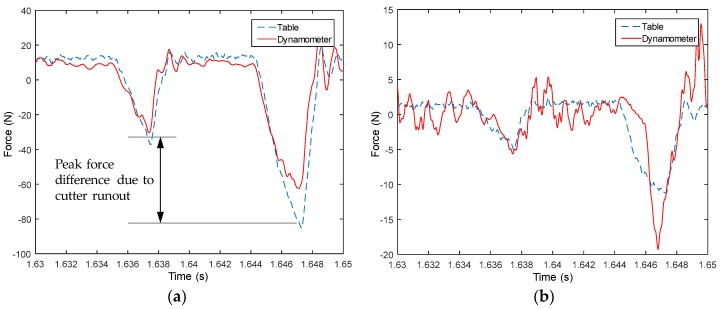
Measured cutting forces for experiments with spindle speed 2000 rpm, feedrate 250 mm/min, axial cutting depth 2 mm and radial cutting depth 1 mm in the (**a**) X direction and (**b**) Y direction.

**Figure 9:**
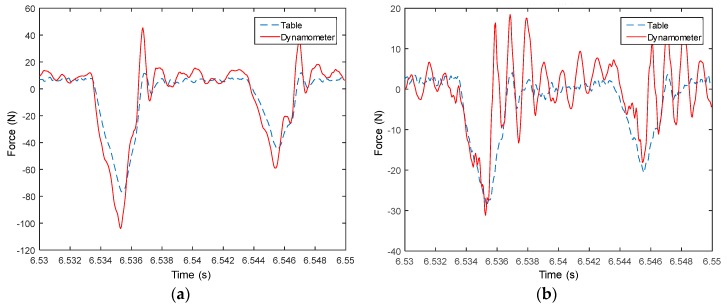
Measured cutting forces for experiments with spindle speed 2000 rpm, feedrate 250 mm/min, axial cutting depth 3 mm and radial cutting depth 2 mm in the (**a**) X direction and (**b**) Y direction.

**Figure 10:**
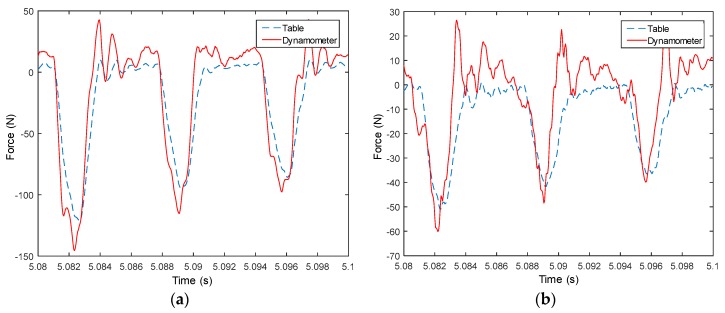
Measured cutting forces for experiments with spindle speed 3000 rpm, feedrate 500 mm/min, axial cutting depth 3 mm and radial cutting depth 3 mm in the (**a**) X direction and (**b**) Y direction.

**Figure 11:**
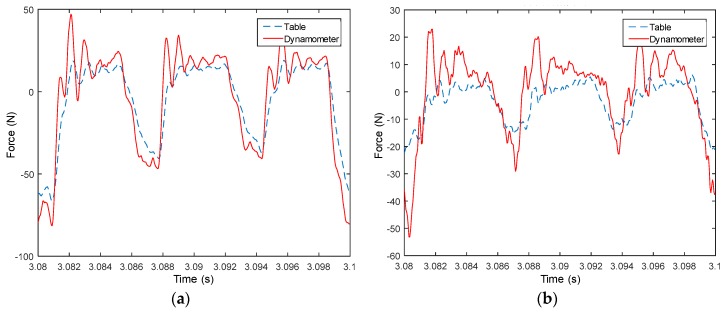
Measured cutting forces for experiments with spindle speed 3000 rpm, feedrate 250 mm/min, axial cutting depth 3 mm and radial cutting depth 3 mm in the (**a**) X direction and (**b**) Y direction.

**Figure 12:**
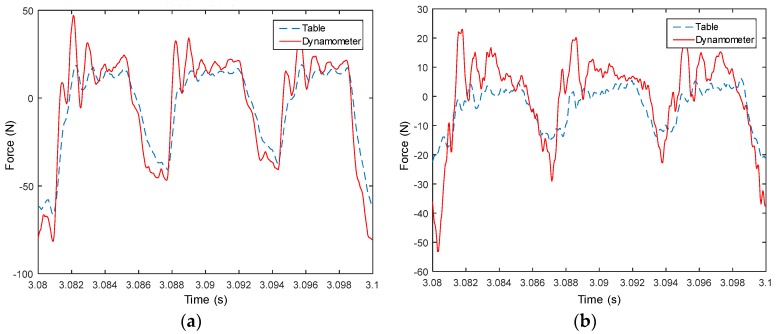
Measured cutting forces for experiments with spindle speed 4000 rpm, feedrate 500 mm/min, axial cutting depth 1 mm and radial cutting depth 3 mm in the (**a**) X direction and (**b**) Y direction.

**Figure 13:**
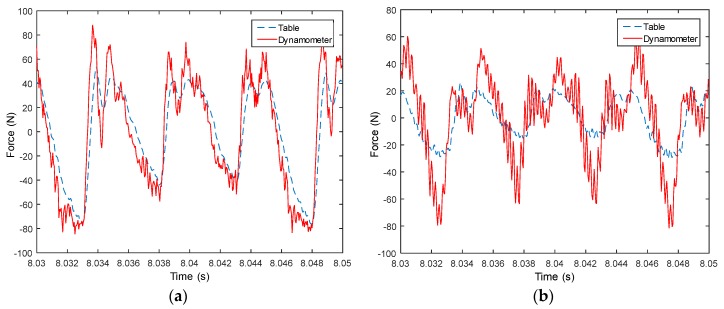
Measured cutting forces for experiments with spindle speed 4000 rpm, feedrate 500 mm/min, axial cutting depth 3 mm and radial cutting depth 3 mm in the (**a**) X direction and (**b**) Y direction.

**Figure 14:**
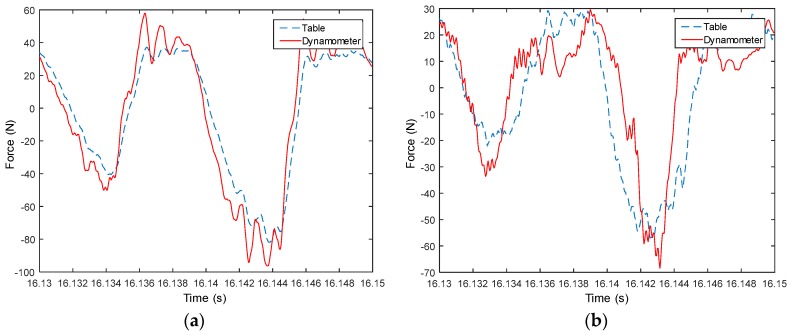
Measured cutting forces for experiments with spindle speed 2000 rpm, feedrate 250 mm/min, axial cutting depth 3 mm and radial cutting depth 3 mm in the (**a**) X direction and (**b**) Y direction.

Measured signals from the table are used to compute forces in both X and Y directions, and then compared with measured forces from the dynamometer, as shown from Figure 8, Figure 9, Figure 10, Figure 11, Figure 12, Figure 13 and Figure 14. Results show that measured forces in the X direction from both the developed instrumented working table and the dynamometer match well in terms of the value, profile and details. In the Y direction, the tendency of both force value and profile are almost the same, except some deviations in the profile for those in Figure 13 and Figure 14. Force signals from the dynamometer in Figure 9 and Figure 13 show different degrees of vibration. These differences and vibration may due to the non-rigid connection between the developed working table and the dynamometer. Besides the force value and profile, the cutter runout in the milling process are well recognized by the developed working table, which is shown by the peak force difference for the neighboring teeth, as shown in those figures.

#### 4.3.2. Force Signal Spectrum Analysis

**Figure 15:**
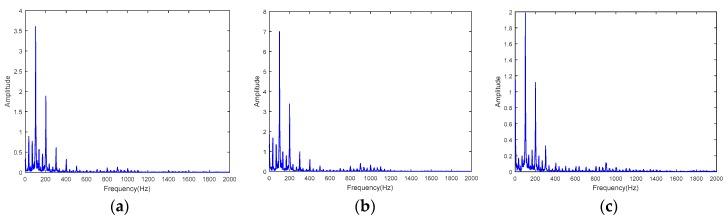
Spectrum analysis results for milling tests with spindle speed 2000 rpm. (**a**) Channel one, (**b**) channel two, and (**c**) channel three.

**Figure 16:**
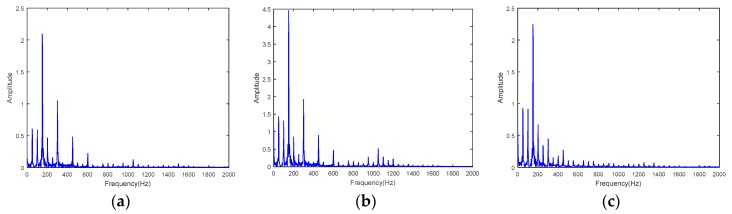
Spectrum analysis results for milling tests with spindle speed 3000 rpm. (**a**) Channel one, (**b**) channel two, and (**c**) channel three.

To further analyze the measured force signals, spectrum analysis is conducted. Shown in Figure 15 are the spectrum analysis results for milling tests with a spindle speed of 2000 rpm. The main frequencies are the multipliers of 100 Hz, which is the tooth passing frequency since a three teeth cutter was used in the tests. For all three channels, the results are almost the same. Spectrum analysis results for milling tests with spindle speed 3000 rpm are shown in Figure 16, the main frequencies are the multipliers of 150 Hz, which is the tooth passing frequency. The force signal spectrum analysis indicates that the developed working table is suitable for milling force measurement. Furthermore, the results also show that the system can capture the high frequency signal portion of the cutting forces.

## 5. Conclusions

A novel instrumented working table used for milling force measurement with embedded PVDF thin-film sensors is proposed, developed and validated in this paper. The main contribution of this paper can be summarized as follows:

(1) PVDF thin-film sensors are integrated into the working table to pick up the dynamic normal strain and to form an instrumented table for milling force measurement.
(2) By applying the force translation theorem and force balance theory, cutting force decoupling models are developed to identify force component in each direction based on the measured signals.
(3) The developed working table is validated by implementation of real milling experiments. Results show that measured forces match well with those from the dynamometer in terms of profile. From the monitored force signals, it can clearly identify the cutter runout in the milling process since the developed working table can capture the force details.
(4) The force signal spectrum analysis indicates that the main frequencies of the measured forces are the multipliers of tool passing frequency, thus the developed working table is suitable for the milling force measurement. Furthermore, the developed system can capture the high frequency signal portion of the cutting forces.

With the developed working table, it can helps to monitor the entire milling process of workpiece, thus enabling monitoring and recognition of the machining condition as well as optimization of the milling process. Further studies on improvement of the instrumented working table as well as the corresponding signal processing method, and the machining condition recognition based on on-line monitored data will be implemented.

## Figures and Tables

**Table 1:** Cutting parameters used in the experiments.

Test No.	Spindle Speed (rpm)	Feedrate (mm/min)	Axial Cutting Depth (mm)	Radial Cutting Depth (mm)
1	2000	250	2.0	1.0
2	2000	250	3.0	2.0
3	2000	250	3.0	3.0
4	3000	500	3.0	3.0
5	3000	250	3.0	3.0
6	4000	500	1.0	3.0
7	4000	500	3.0	3.0
